# Modelling cointegration and Granger causality network to detect long-term equilibrium and diffusion paths in the financial system

**DOI:** 10.1098/rsos.172092

**Published:** 2018-03-28

**Authors:** Xiangyun Gao, Shupei Huang, Xiaoqi Sun, Xiaoqing Hao, Feng An

**Affiliations:** 1 School of Humanities and Economic Management, China University of Geosciences, Beijing 100083, People's Republic of China; 2 Key Laboratory of Carrying Capacity Assessment for Resource and Environment, Ministry of Land and Resources, Beijing 100083, People's Republic of China; 3 Open Lab of Talents Evaluation, Ministry of Land and Resources, Beijing 100812, People's Republic of China

**Keywords:** complex network, time series, cointegration, Granger causality, financial system

## Abstract

Microscopic factors are the basis of macroscopic phenomena. We proposed a network analysis paradigm to study the macroscopic financial system from a microstructure perspective. We built the cointegration network model and the Granger causality network model based on econometrics and complex network theory and chose stock price time series of the real estate industry and its upstream and downstream industries as empirical sample data. Then, we analysed the cointegration network for understanding the steady long-term equilibrium relationships and analysed the Granger causality network for identifying the diffusion paths of the potential risks in the system. The results showed that the influence from a few key stocks can spread conveniently in the system. The cointegration network and Granger causality network are helpful to detect the diffusion path between the industries. We can also identify and intervene in the transmission medium to curb risk diffusion.

## 1. Introduction

The financial stock system is formed by lots of stocks, which belong to different industries. The financial market is a complex and chaotic system, in which a slight move in one part may affect the situation as a whole. As we all know, the subprime crisis in the USA caused the global economic crisis [1,2], because real estate is a key industry in the national economy with characteristics of a long industry chain, value chain and large demand of financing [3–5]. The real estate industry (REI) has a driving effect, which has a great impact on other industries. The driving effect of REI attracts the attention of all sectors in society. Governments can intervene in REI by financial policy for promoting macroeconomic stability and equilibrium development [6–9]. On the other hand, shocks to REI may spread risk in the financial and economic system, which comprises the REI and its upstream and downstream industries. Changes in one industry also may cause different policies to be made or updated. For example, to avoid a financial bubble in REI, China has enacted a series of policies restricting the purchase of houses, particularly in megacities such as Beijing. Each family in a first-tier city of China can only buy a strictly limited number of houses, which has even led to the farce of fake divorces occurring to buy more houses. Hence, it is necessary to study the relationship between different industries to understand the mechanism of the potential risk proliferation in the financial system.

Previous studies have demonstrated that there exists a co-movement and causality between the housing and stock markets [10–13]. Most current studies have focused on the issues of the impact of the REI on other industries in the national economy from a macroscopic perspective or at the industry level [14,15]. Two approaches have been widely used in this issue from a systematic perspective: the input–output analysis method based on industry linkage theory and the computable general equilibrium (CGE) model. Researchers examined the role of the REI in the national economy based on input–output tables, and they found that real estate had a great impact on other sectors [14,16]. Based on CGE, researchers established a dynamic equilibrium model of urban housing for investigating the issue of taxes and allowances [17]. The dynamic CGE model of China's real estate and macro-economy were developed for simulating the policy effects [18]. Summarizing the above, the input–output analysis method and CGE model provide practical approaches for studying the industry linkages of real estate.

However, both approaches have some limitations, i.e. data are updated slowly, and data demand is complex. Because of the limitations, some results also face the limitation of timeliness. In particular, in the financial system, the responses between the stock markets of industries (such as REI, steel industry (SI), building materials industry (BMI), construction products and engineering industry (CPEI), banking industry (BI) and the household durable consumer goods industry (HDCGI)) are rapid. Thus, in this paper, we use modern econometric methods to detect the relationships between the stock markets of the different industries based on real-time stock price data.

Another important issue for understanding the financial stock system is to understand the macrobehaviour from a microstructure perspective. The input–output analysis method and CGE model have shown the advantage of the core idea from micro to macro, which is clearly to identify the contribution of each industry to the entire economic system [19,20]. However, most of the current literature has focused on the macro or industry perspective separately. In fact, there are lots of listed companies in every industry in the financial stock system. If we study the linkages between these industries from the microstructure perspective of the relationships between these listed companies, it will provide more detailed information for understanding the financial stock system.

Through this kind of research paradigm (from microstructure to macrobehaviour), obviously, the relationships between the listed companies form a linked network. Thus, we can establish and analyse the network model to understand the financial stock system. Fortunately, complex network theory, as a hot topic, currently provides an approach for network modelling and analysis based on time series [21–29]. The core idea of complex network theory is that the structure determines the function of the system [30]. There is much in the literature that models and analyses the financial networks based on complex network theory [31–36]. They studied the role of different types of listed companies and the risk diffusion issue in the stock market, and they quantify the relationship between listed companies based on the stock prices [33,37–40]. However, in most of the current literature, the definition of relationships between stocks prices are simple correlations based on cross-correlation coefficients or partial correlation coefficients [41]. The cointegration and Granger causality test should be considered for modelling the stock complex network, which can reveal the long-term equilibrium and the causality direction of the diffusion paths in the financial stock system.

Hence, we used complex network theory to visualize the linked network between the listed companies of different industries in the financial stock system. We collected the daily stock price time series data of the REI and other correlative upstream and downstream industries, which involved hundreds of stocks. The relationships between stock prices were quantified by econometric methods. Then, we constructed and analysed the financial stock complex network models from two aspects: the cointegration network model and the Granger causality network model. Thereby, we provide insights for policy decisions and risk diffusion prevention.

## 2. Material and methods

### 2.1. Materials

To study the relationships in the financial stock system based on the network analysis paradigm, we used the stock price time series for building network models. Here, we selected the stock closing prices for the REI, SI, BMI, CPEI, BI and HDCGI in the China stock market as sample data. There was a total of 309 stock price time series. Each stock price time series contains 200 daily data points in 2016. All data come from the Wind Economic Database.

The statistics of the industries are summarized in table 1. The level of stock prices is different in different industries. In the same industry, there is also wide variation between different stocks. (i) There are 120 stocks in REI, the mean stock price is 11.14 CNY and the minimum stock price is 1.08 CNY, but the maximum stock price is 67.60 CNY. (ii) The SI, which plays an important role in the REI, is facing the difficult situation of overcapacity in China. The mean stock price of the 37 stocks in SI is only 8.16 CNY, and the median stock price is 5.74 CNY. The slowdown of future economic growth and real estate development would cause the China SI a bleak future. (iii) The stock prices of BMI and CPEI are at similar levels. The REI affects the BMI and the CPEI directly. (iv) There is a close relationship between the REI and the BI in China. The cumulative amount of bank loans reached 14 795 trillion CNY in the funds sources of real estate development investment from February to October in 2016 (data from the QIANZHAN database; http://d.qianzhan.com/). The China BI has fallen into an embarrassing situation, which bears the risk from the REI. (v) For the downstream industry of the REI, HDCGI, the mean stock price of HDCGI is 23.08 CNY, which is higher than that of other industries. The REI affects the HDCGI indirectly by the home improvement demand from house buyers.

**Table 1.** The statistics of stock prices of different industries (CNY).

industry	*N*	mean	median	minimum	maximum
REI	120	11.14	9.29	1.08	67.60
SI	37	8.16	5.74	2.29	54.38
BMI	34	11.70	8.84	1.05	90.10
CPEI	86	13.59	10.82	3.57	84.18
BI	16	9.19	9.13	2.89	18.75
HDCGI	16	23.08	18.97	1.42	68.00

### 2.2. Methods

**Figure 1.**
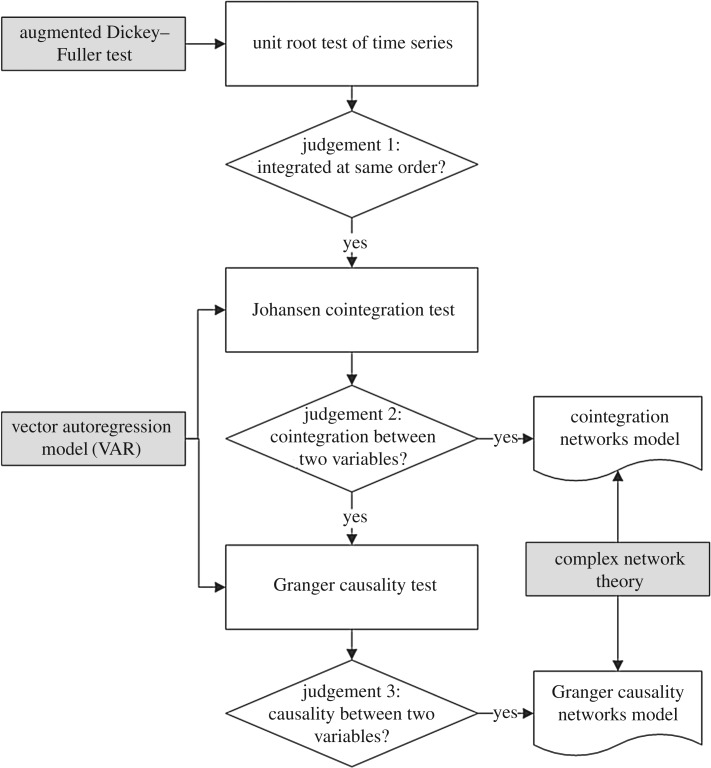
The process of cointegration network model and Granger causality network model building.

First, we built the network models for different research issues. For example, to study the long-term equilibrium relationships in the financial stock system, we built the cointegration network model combined with econometric models and complex networks theory. Then, based on the cointegration network model, we built the Granger causality network model for understanding the causality direction in the financial stock system. The process of network models building is shown in figure 1, and the detailed steps are as follows.

#### 2.2.1. Step 1: unit root test of univariate time series

The necessary condition of the cointegration test is the integration at the same order between different variables. Thus, we tested the integration order of each variable time series by the augmented Dickey–Fuller test method. If there is the same order of integration between two variables, we can do the cointegration test.

#### 2.2.2. Step 2: cointegration test

A cointegration relationship between two variables means that their linear combination is stationary. Some researchers have established the stock complex network based on the cointegration test [42,43]. However, they used the Engle–Granger method for the cointegration test. The Engle–Granger method is based on the residual test, but the Johansen cointegration test is based on the regression parameters test. The Engle–Granger method assumes that one variable is endogenous and the other is exogenous, which does not allow for the possible endogeneity of the regressors. But the Johansen cointegration test allows all variables to be endogenous. And the Johansen cointegration test has greater power than the Engle–Granger method [44,45]. Thus, we can use the Johansen cointegration test method based on the vector autoregression (VAR) model for testing the cointegration relationship between variables.

It should be noted that we test the cointegration relationship between two variables in this paper. Because there are 309 variables in the financial stock system, considering hundreds of variables in one VAR model is unrealistic. The simplest and most basic elements of the intricate relationships among hundreds of variables in the financial stock system are the relationships between two variables. Thus, we tested the cointegration relationship between two variables in the 309 variables, which means that we should build $(309^{2}-309)\text{/}2=47\;586$ VAR models for the Johansen cointegration tests with the 47 586 times. The VAR(*p*) model between two variables $Y_{t}^{i}$ and $Y_{t}^{j}$ is as follows:

2.1 $$\left(\begin{array}{c} Y_{t}^{j}\\ Y_{t}^{i} \end{array}\right)=\Phi_{1}\left(\begin{array}{c} Y_{t-1}^{j}\\ Y_{t-1}^{i} \end{array}\right)+\Phi_{2}\left(\begin{array}{c} Y_{t-2}^{j}\\ Y_{t-2}^{i} \end{array}\right)+\cdots +\Phi_{p}\left(\begin{array}{c} Y_{t-p}^{j}\\ Y_{t-p}^{i} \end{array}\right)+\epsilon_{t},$$

where $Y_{t}^{i}$ and $Y_{t}^{j}$ indicate two stock closing prices time series; $\Phi_{p}$ is the matrix of coefficients of 2 × 2 dimensions; $\epsilon_{t}$ is the column vector of stochastic error of two dimensions; *p* is the lag order and the value of *p* is selected by Akaike Information Criterion and Schwarz Criterion. We selected the smaller one of the lag orders as the value of *p* from the results of two criteria based on the 5% significance level. Thus, the value of *p* can not only meet the requirement of the model setting but also avoid the excessive losses of the freedom degrees.

Based on the VAR(*p*) models, we conducted the Johansen cointegration tests between two variables through a trace test of the characteristic roots. Then, we judged the cointegration relationships by comparing with the Johansen distribution critical values. If the trace statistical values of the characteristic roots are greater than the Johansen distribution critical values under the 5% significance level, there exists cointegration vectors, which means that there are significant cointegration relationships between variables.

#### 2.2.3. Step 3: the cointegration network model building

To build the cointegration network model, we should define the vertexes and the edges. In this paper, we defined the stocks as vertexes and the significant cointegration relationships between stocks as edges. The cointegration network model of the financial stock system can be indicated by an upper triangular matrix $G_{\mathrm{cointegration}}$.

2.2 $$G_{\mathrm{cointegration}}=(V_{\mathrm{cointegration}},E_{\mathrm{cointegration}})=\left[\begin{array}{cccc} 0 & \text{coin}{\text{t}}_{1,2} & \ldots & \text{coin}{\text{t}}_{1,k}\\ 0 & 0 & \ldots & \text{coin}{\text{t}}_{2,k}\\ \vdots & \vdots & \ddots & \vdots \\ 0 & 0 & \ldots & 0 \end{array}\right],$$

where $V_{\mathrm{cointegration}}$ is the vertex set, $E_{\mathrm{cointegration}}$ is the edge set and $\text{coin}{\text{t}}_{i,j}$ indicates whether there is a significant cointegration relationship between stock *i* and stock *j*.

2.3 $$\text{coin}{\text{t}}_{i,j}=\left\{ \begin{array}{ll} 1 & \text{there is significant cointegration relationship}\\ 0 & \text{there is no significant cointegration relationship}. \end{array}\right.$$

**Figure 2.**
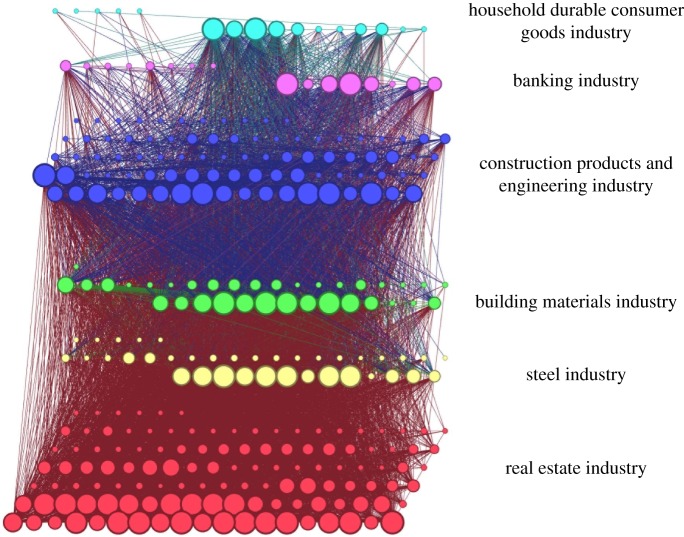
The cointegration network model of the financial stock system. (Vertex with a greater degree is shown larger. Different layers represent different industries.)

Thus, the cointegration network is an undirected and unweighted complex network, which is visualized by the network graph, as shown in figure 2. Owing to the structure of complex networks determining the function of the system [30], we can understand the long-term equilibrium relationship characteristics in the financial stock system by studying the cointegration network.

#### 2.2.4. Step 4: Granger causality test

The essence of the Granger causality test is to test whether a lag variable can be introduced into other variable equations. If one variable is affected by the lag of other variables, there is Granger causality between them. Thus, we tested Granger causality between two variables in the financial stock system, and we defined that there exists Granger causality between two stocks if the Granger causality tests pass the significance test under the 5% level.

#### 2.2.5. Step 5: the Granger causality network model building

Risk diffusion is similar to the spread of a virus, which needs a platform for diffusion. The relationship network is the platform. In the financial stock system, the fluctuation from any industry may have an impact on other industries, even on the entire system. The risks may be produced by the fluctuations; hence, the fluctuations or the risks may diffuse in the system based on the Granger causality network. However, due to the different structures of networks determining different risk diffusion forms in the system, it is necessary to understand the structure of the Granger causality network.

In this paper, we defined the stocks as vertexes and the significant Granger causality between stocks as edges (the significant level is 5%). The Granger causality network model of the financial stock system can be indicated by an adjacency matrix $G_{\mathrm{causality}}$.

2.4 $$G_{\text{causality}}=(V_{\text{causality}},E_{\text{causality}})=\left[\begin{array}{cccc} 0 & {\text{caus}}_{1,2} & \ldots & {\text{caus}}_{1,k}\\ {\text{caus}}_{2,1} & 0 & \ldots & {\text{caus}}_{2,k}\\ \vdots & \vdots & \ddots & \vdots \\ {\text{caus}}_{k,1} & {\text{caus}}_{k,2} & \ldots & 0 \end{array}\right],$$

where $V_{\mathrm{causality}}$ is the vertex set, $E_{\mathrm{causality}}$ is the edge set and $\text{cau}{\text{s}}_{i,j}$ indicates whether stock *i* Granger causes stock *j* significantly.

2.5 $$\text{cau}{\text{s}}_{i,j}=\left\{ \begin{array}{ll} 1 & \text{stock }i\text{ Granger causes stock }j\text{ significantly}\\ 0 & \text{there is no significant Granger causality}. \end{array}\right.$$

**Figure 3.**
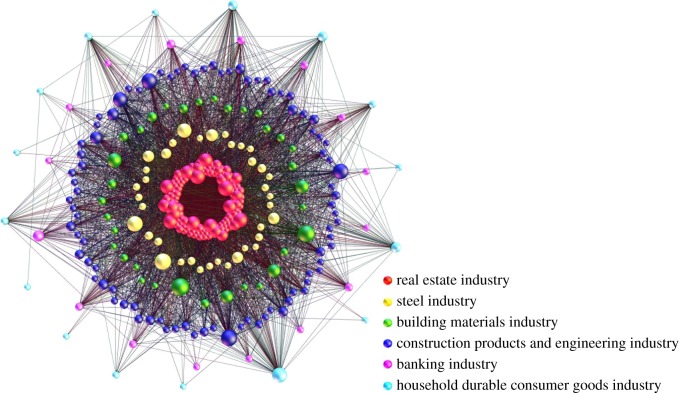
The Granger causality network model of the financial stock system. (Vertex with a greater out-degree is shown larger. Different circles represent different industries.)

The Granger causality is directional; thus, the Granger causality network is a directed and unweighted complex network, which is visualized by network graph, as shown in figure 3.

## 3. Results

The network analysis paradigm is that we can study the relationships in the financial stock system by analysing the structures of the network models. The networks provide the platforms for the interaction behaviours between industries. Hence, we analysed two types of networks (cointegration network model and Granger causality network model) for understanding the interaction behaviours in the financial stock system. For the cointegration network model analysis, we can detect the long-term equilibrium relationships in the financial stock system. For the Granger causality network model analysis, we can identify the transmission directions and the diffusion paths of the fluctuation or the potential risks.

### 3.1. Cointegration network model analysis

The purpose of the cointegration test is to identify the long-term equilibrium relationships between variables. To understand the long-term equilibrium relationships in the financial stock system, we analysed three aspects: the basic statistical analysis of the cointegration network, the degree analysis of the cointegration network and the cointegration analysis between industries.

#### 3.1.1. Basic statistical analysis of the cointegration network

We tested the cointegration relationships between two variables in 309 stock price time series (vertexes). The visualization of the cointegration network is shown in figure 2, and the statistical results of the indicators of the cointegration network analysis are shown in table 2.

**Table 2.** The statistic of the indicators of cointegration network analysis.

indicators	values
number of vertexes	309
number of edges	8304
density of network	0.175
diameter of network	7
average path length of network	2.093

Theoretically, the maximum number of the cointegration relationships is $(309^{2}-309)/2=47\;586$; however, there are only 8304 significant cointegration relationships (edges) in the financial stock system. The density of the cointegration network is 0.175. Most of the relationships (1−0.175=82.5%) between stocks are not cointegration. It means that although there is a close correlation between different industries, the long-term equilibrium relationships are sparse from the microstructure perspective. However, the average path length of the network is only 2.093, and the diameter of the network is 7, which means that the connectivity of the cointegration network is good. The fluctuation of any variable can spread conveniently based on the cointegration network in the system.

Previous studies showed that there existed cointegration between industries from the macroscopic perspective [10,14], but the cointegration did not always exist from the microscopic perspective. Thus, sometimes, we are easily confused by superficial phenomena from the macro perspective. In the financial stock system, enterprises and the relationships between enterprises are the microstructure basis of the macrosystem. It is necessary to understand the microstructure of the cointegration network for obtaining more detail of the relationships between industries. It is important to study the role of each stock in the cointegration network.

#### 3.1.2. Degree analysis of the cointegration network

Degree analysis can help us understand the role of each stock in the cointegration network. Because some key stocks play important roles in the financial stock system, the key stocks have many long-term equilibrium relationships with other stocks. When one of the key stock fluctuates drastically, many other stocks may be affected. To identify the key stocks, we measure the degree of the vertexes. The degree of one vertex could be considered as the number of the edges with other adjacent vertexes. The degree of the vertex *i* is defined as *D_i_*

3.1 $$D_{i}=\underset{j\in N_{i}}{\sum }\text{coin}{\text{t}}_{i,j},$$

where *N_i_* is the set of adjacent vertexes of the vertex *i*, and $\text{coin}{\text{t}}_{i,j}$ is the value (0 or 1) between vertex *i* and vertex *j*. A stock (vertex) with a greater degree is shown larger in figure 2, and it has greater importance in the financial stock system.

The average degree of the cointegration network is defined as $\bar{D}$

3.2 $$\bar{D}=\frac{\sum_{i=1}^{n}D_{i}}{N},$$

where *N* is the number of vertexes in the cointegration network.

The average degree of each industry in the cointegration network is defined as ${\bar{D}}_{k}$

3.3 $${\bar{D}}_{k}=\frac{\sum_{i\in K}D_{i}}{N_{k}},$$

where *K* is the vertexes set of one industry, and *N_k_* is the number of vertexes in the industry. There are six industries in the financial stock system in this paper. Thus, we can measure the importance of each industry by ${\bar{D}}_{k}$. For an industry with a greater average degree, the greater importance it has in the financial stock system.

**Figure 4.**
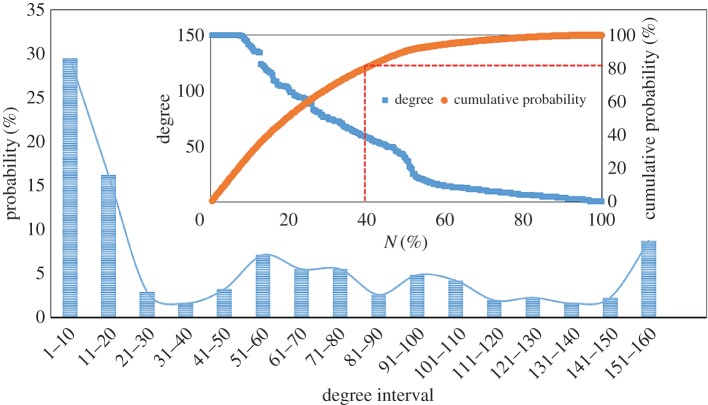
Degree distribution of the cointegration network.

The degree distribution is neither a normal distribution nor a power-law distribution. The average degree of the cointegration network is 53.75, which means that each stock has cointegration relationships on average with approximately 54 other stocks. The degree distribution of the cointegration network is shown in figure 4. We can find that the degrees of nearly 30% of the stocks are between 1 and 10, the degrees of 53.40% of the stocks are less than 50 and the degrees of 21.04% of the stocks are more than 100. We sorted all the stocks by the degrees in descending order, and then we calculated the cumulative probability, as shown in the inset graph of figure 4. Only 39.48% of the stocks are responsible for more than 80% of the cumulative probability.

The results of the degree distribution show the situation of the cointegration relationships in the financial stock system. A few stocks with large degrees play important roles in the system, but most stocks have small degrees (for the degree of each stock, see electronic supplementary material, table S1).

The degree statistic of each industry in the cointegration network is shown in table 3. From table 3, we can find that the BMI and REI play the most important roles in the financial stock system. The average degrees of CPEI, HDCGI and SI are lower than other industries in the system. The average degree of BI is in the middle. In the financial stock system, there is not always cointegration between two stocks in the total 309 stocks. One stock has at least one cointegration relationship with other stocks and at most 155 cointegration relationships with other stocks.

**Table 3.** The degree statistic of each industry.

industry	average degree	maximum degree	minimum degree	s.d. of degree
REI	60.03	155	5	51.33
SI	46.57	155	3	51.73
BMI	61.00	155	1	46.77
CPEI	46.41	150	1	53.70
BI	54.56	155	1	53.61
HDCGI	46.44	151	1	52.13

#### 3.1.3. Cointegration analysis between industries

The cointegration analysis between industries is different from modern econometric analysis methods in this paper. We did not test the cointegration relationships between industries by the macroscopic industry indexes series. Because there are hundreds of stocks in the system, we counted the number of the cointegration relationships between different industries. Thus, the cointegration between industries (macroscopic level) is reflected by cointegration between stocks (microscopic level). We defined the extent of cointegration between industry *I* and industry *J* as $C_{I-J}$, and the equation is as follows:

3.4 $$C_{I-J}=\frac{N(\text{Coi}{\text{n}}_{I-J})}{N(I)*N(J)},\;I\neq J,$$

where $N(\text{Coi}{\text{n}}_{I-J})$ is the number of the cointegration relationships between industry *I* and industry *J*, and *N*(*I*) and *N*(*J*) are the number of the stocks in industry *I* and industry *J*, respectively. *N*(*I*) × *N*(*J*) means the theoretical maximum number of the cointegration relationships between industry *I* and industry *J*. $C_{I-J}\in [0,1]$ and $C_{I-J}=C_{J-I}$. $C_{I-J}$ with a greater value leads to the greater extent of cointegration between industries in the financial stock system.

The extent of cointegration between industries is shown in table 4. There exists cointegration between different industries, but the extent of cointegration is different. The top three of the extent of cointegration are REI–BMI, REI–BI and BMI–BI. It means that there exist steady equilibrium relationships among the REI, BMI and BI.

**Table 4.** The extent of cointegration between industries.

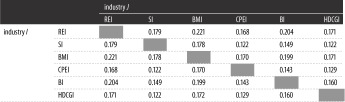

The results from table 4 are different from current studies [10,12,14]. As we mentioned above, microscopic factors are the basis of macroscopic phenomena. Although much evidence can indicate the long-term equilibrium relationships between REI and other correlative industries, the perspectives of these studies were macroscopical. The equilibrium relationships between stocks of the REI and other correlative industries do not always exist. Thus, table 4 provides the extent of cointegration between different industries from the micro perspective.

### 3.2. Granger causality network model analysis

The Granger causality can reflect whether one time series variable is useful in forecasting another. When one time series variable Granger causes another time series variable, the fluctuant patterns in the variable are approximately repeated in the other variable after some time lag. The Granger causalities between many variables form a network in a system. The fluctuation from any variable may diffuse in the system based on the Granger causality network. Thus, we can study the transmission directions and the diffusion paths of the fluctuation or the potential risks by analysing the Granger causality network model. In this part, we analysed four aspects of the Granger causality network model: the basic statistical analysis of the Granger causality network, in-degree and out-degree analysis of the Granger causality network, the identification of the transmission medium and the Granger causality analysis between industries.

#### 3.2.1. Basic statistical analysis of the Granger causality network

Granger causality network is a directed and unweighted complex network, as shown in figure 3. The statistical results of the indicators of the Granger causality network analysis are presented in table 5. There are 6034 significant Granger causality (edges) in the financial stock system. The density of the Granger causality network is 0.063. Compared with the cointegration network, the Granger causality is sparser.

**Table 5.** The statistics of the indicators of Granger causality network analysis.

indicators	values
number of vertexes	309
number of edges	6034
density of network	0.063
diameter of network	8
average path length of network	2.609

The results reflect that the number of diffusion paths is limited. However, the diameter of the Granger causality network is 8, and the average path length of the Granger causality is only 2.609, which means that the diffusion is fast and convenient. The fluctuation or the potential risks can diffuse through the entire system by only a few stocks (transmission medium). In the platform of the Granger causality network, different stocks or industries play different roles in the diffusion.

#### 3.2.2. In-degree and out-degree analyses of the Granger causality network

In-degree and out-degree analyses can help us understand the role of each stock or each industry in the diffusion process based on the Granger causality network. Because the Granger causality network is a directed complex network, we consider the direction of the Granger causality. Some stocks may Granger cause other stocks, and conversely, some stocks may be Granger caused by other stocks. To calculate the number of the stocks that a stock Granger causes, we measure the out-degree of the stock (vertex). To calculate the number of the stocks that a stock is Granger caused, we measure the in-degree of the stock (vertex). The in-degree of one vertex could be considered as the number of the edges to the vertex, and the out-degree of one vertex could be considered as the number of the edges to other vertexes. The in-degree of the vertex *i* is defined as $D_{i}^{\mathrm{in}}$, and the out-degree of the vertex *i* is defined as $D_{i}^{\mathrm{out}}$.

3.5 $$D_{i}^{\mathrm{in}}=\underset{j\in N_{i}}{\sum }\text{cau}{\text{s}}_{i\leftarrow j}$$

and

3.6 $$D_{i}^{\mathrm{out}}=\underset{j\in N_{i}}{\sum }\text{cau}{\text{s}}_{i\to j},$$

where *N_i_* is the set of adjacent vertexes of the vertex *i*, $\text{cau}{\text{s}}_{i\leftarrow j}$ is the value (0 or 1) from vertex *j* to vertex *i*, and $\text{cau}{\text{s}}_{i\to j}$ is the value (0 or 1) from vertex *i* to vertex *j.* A stock (vertex) with greater out-degree is shown larger in figure 3, and the more other stocks are Granger caused by the stock, the greater importance it has in the diffusion process of the financial stock system. A stock (vertex) with greater in-degree is easier to be affected by other stocks and more sensitive in the system.

Because the Granger causality is paired, the average in-degree $\bar{D^{\mathrm{in}}}$ is equal to the average out-degree $\bar{D^{\mathrm{out}}}$ of the Granger causality network.

3.7 $$\bar{D^{\mathrm{in}}}=\frac{\sum_{i=1}^{n}D_{i}^{\mathrm{in}}}{N}=\bar{D^{\mathrm{out}}}=\frac{\sum_{i=1}^{n}D_{i}^{\mathrm{out}}}{N},$$

where *N* is the number of vertexes in the Granger causality network.

The average in-degree of each industry in the Granger causality network is defined as ${\bar{D^{\mathrm{in}}}}_{k}$, and the average out-degree of each industry in the Granger causality network is defined as ${\bar{D^{\mathrm{out}}}}_{k}$.

3.8 $${\bar{D^{\mathrm{in}}}}_{k}=\frac{\sum_{i\in K}D_{i}^{\mathrm{in}}}{N_{k}}$$

and

3.9 $${\bar{D^{\mathrm{out}}}}_{k}=\frac{\sum_{i\in K}D_{i}^{\mathrm{out}}}{N_{k}},$$

where *K* is the vertexes set of one industry and *N_k_* is the number of vertexes in the industry. We can measure the importance of each industry by ${\bar{D^{\mathrm{in}}}}_{k}$ and ${\bar{D^{\mathrm{out}}}}_{k}$. For an industry with greater average out-degree, more industries are Granger caused by the industry, and the greater importance it has in the financial stock system. An industry with greater average in-degree is easier to be impacted by other industries and more sensitive in the system.

**Figure 5.**
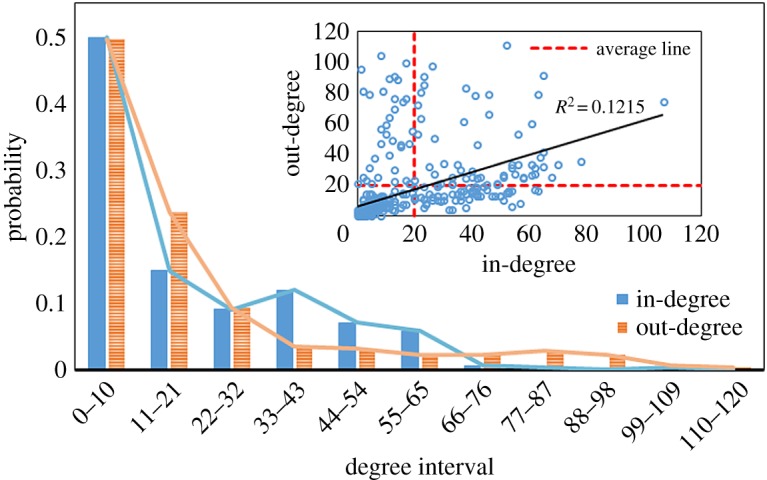
In-degree and out-degree distributions of the Granger causality network.

The in-degree and out-degree distributions both follow long-tail distributions. As shown in figure 5, we find that the probability of the in-degree and out-degree declines rapidly with an increase in the degree interval. The in-degree and out-degree of nearly half of the stocks are between 1 and 10. Only a few of the stocks play a key driving role in the diffusion process in the financial stock system. However, because of the long-tail distributions, these key stocks are the Granger causes of hundreds of other stocks, and the fluctuation from key stocks may have an impact on the entire system. Thus, we should pay more attention to these key stocks for risk monitoring.

We also plot the distribution between in-degree and out-degree, as shown in the inset graph of figure 5. The average line indicates the average in-degree and the average out-degree. The average in-degree or out-degree of the Granger causality network is 19.53. There is a weak positive correlation between in-degree and out-degree, for which *R*^2^ = 0.1215. It means that while the stocks influence other stocks, the stocks are also easily influenced by other stocks. Four quadrants are divided by the average lines that can help us identify the types of stocks in the system. There are 58 stocks (vertexes) in the first quadrant (such as Beijing Capital Development Holding), 35 stocks in the second quadrant (such as Bank of China), 159 stocks in the third quadrant and 57 stocks in the fourth quadrant (such as China Construction Bank). Different quadrants mean different types of stocks. For example, the stocks of the second quadrant influence many other stocks, but they are not easily influenced by others. The situation of the stocks of the third quadrant is contrary with the first quadrant (for the in-degree and out-degree of each stock, see electronic supplementary material, table S1).

The degree statistic of each industry in the Granger causality network is shown in table 6. The BMI and the REI have larger out-degrees, which play the most important roles in the financial stock system. At the same time, both industries with larger in-degrees are also easily influenced by other industries. Comparing the in-degree and the out-degree, the SI influences other industries more easily. In particular, China is experiencing the reform of reducing the production capacity in the SI. The fluctuation of the SI may cause the fluctuation of the REI and other industries. Conversely, the BI is more easily influenced by other industries. The fund of the REI mainly depends on the bank loans in China [46]. Hence, in fact, the risk is passed on to the BI. The benefits of other industries are directly related to the risk of the BI. This is also a thorny problem for China's real estate finance.

**Table 6.** The in-degree and out-degree statistic of each industry.

	in-degree				out-degree			
industry	average	maximum	minimum	s.d.	average	maximum	minimum	s.d.
REI	21.67	107	0	21.05	21.98	95	0	25.01
SI	12.68	54	0	13.14	20.00	99	0	25.73
BMI	23.12	63	0	20.92	23.91	111	0	29.60
CPEI	18.06	70	0	19.81	16.30	97	0	20.12
BI	19.19	53	2	16.92	12.44	47	1	11.22
HDCGI	19.94	54	1	19.89	15.19	83	0	21.60

#### 3.2.3. Identification of the transmission medium

**Figure 6.**
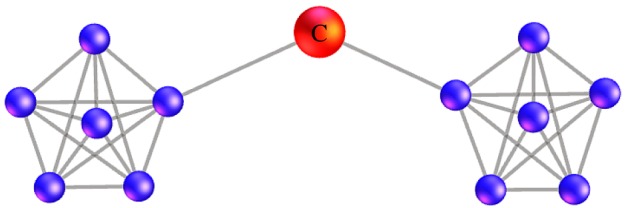
A sample of the transmission media in a network.

Similar to the spread of a virus, we focus not only on the key stocks (vertexes) with a large out-degree or in-degree but also on the transmission medium in the diffusion process. Each vertex can play an intermediary role in a network. However, different vertexes have different intermediary abilities. Figure 6 shows a sample of the transmission media in a network. Although the degree (in-degree or out-degree) of the vertex *C* is small, it has a strong intermediary ability. If we identify the transmission medium in the Granger causality network, it is helpful to curb the diffusion of the adverse risks.

Thus, we can evaluate the intermediary ability by calculating the normalized betweenness centralities of a vertex. The normalized betweenness centrality of vertex *i* can be defined as BC*_i_*

3.10 $$\text{B}{\text{C}}_{i}=\frac{\sum_{j}^{n}\sum_{k}^{n}g_{jk}(i)\text{/}g_{jk}}{N^{2}-3N+2},\;j\neq k\neq i,\;j<k,$$

where *g_jk_*(*i*) is the number of shortest paths between vertex *j* and vertex *k* that pass vertex *i*. *g_jk_* is the total number of shortest paths between vertex *j* and vertex *k*. A stock (vertex) with greater normalized betweenness centrality has stronger intermediary ability in the system.

We can also measure the intermediary ability of each industry in the Granger causality network by calculating the average normalized betweenness centrality of industry. The average normalized betweenness centrality of each industry in the Granger causality network is defined as ${\bar{\text{BC}}}_{k}$

3.11 $${\bar{\text{BC}}}_{k}=\frac{\sum_{i\in K}\text{B}{\text{C}}_{i}}{N_{k}}.$$

An industry with greater average normalized betweenness centrality has stronger intermediary ability in the financial stock system.

**Figure 7.**
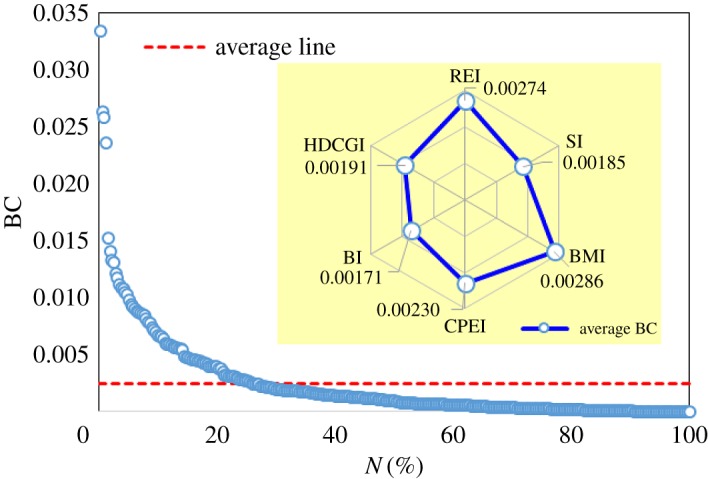
The distribution of the normalized betweenness centrality.

The distribution of the normalized betweenness centrality in the Granger causality network is shown in figure 7. The average line indicates the average normalized betweenness centrality. The average normalized betweenness centrality of the Granger causality network is 0.00243. Only 25.89% of the stocks are above the average line, and most of the stocks (74.11%) have low normalized betweenness centrality. It indicates that a few stocks play an important intermediary role in the process of risk diffusion.

We also plot the average normalized betweenness centrality of each industry in the inset graph of figure 7. The REI and BMI have stronger intermediary abilities in the financial stock system. However, the BI and SI have weaker intermediary abilities. When risk occurs, it is helpful to understand how much intermediary ability each industry has in the process of risk diffusion. We should pay more attention to industries with strong intermediary abilities.

#### 3.2.4. Granger causality analysis between industries

For measuring the extent of the Granger causality between industries, we count the number of the Granger causality between different industries. Similar to the cointegration analysis between industries, the Granger causality between industries (macroscopic level) is reflected by Granger causality between stocks (microscopic level). Because the Granger causality is directional, we defined that the extent of industry *I* Granger causes industry *J* as $\text{G}{\text{C}}_{I\to J}$, and the equation is as follows:

3.12 $$\text{G}{\text{C}}_{I\to J}=\frac{N(\text{Cau}{\text{s}}_{I\to J})}{2*N(I)*N(J)},\;I\neq J,$$

where $N(\text{Cau}{\text{s}}_{I\to J})$ is the number of the Granger causality between industry *I* and industry *J*, and N(I) and N(J) are the number of the stocks in industry *I* and industry *J*, respectively. The term 2∗N(I)∗N(J) means the theoretical maximum number of the Granger causality between industry *I* and industry *J*. $\text{G}{\text{C}}_{I\to J}\in [0,1]$. $\text{G}{\text{C}}_{I\to J}$ with a greater value has a greater extent of Granger causality between industries in the financial stock system.

**Figure 8.**
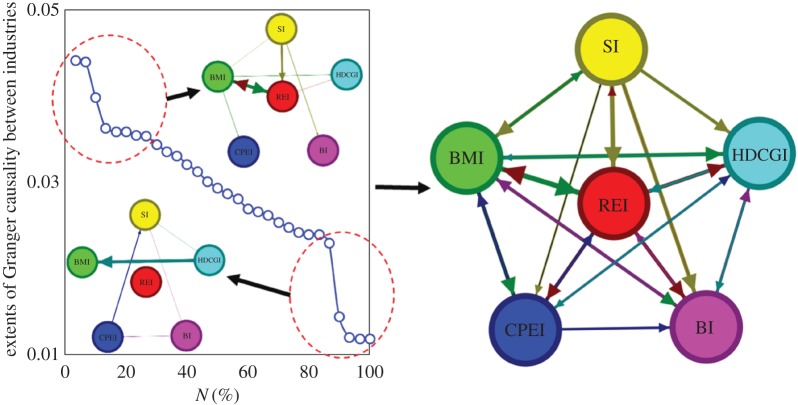
The Granger causality between industries under different thresholds. (The thicker an edge between vertexes, the stronger is the extent of Granger causality between industries.)

The extent of Granger causality between industries is shown in table 7. There exists bidirectional Granger causality between different industries, but the extent of Granger causality is different. We visualize the Granger causality between industries under different thresholds of the extent, as shown in figure 8.

**Table 7.** The extent of Granger causality between industries.

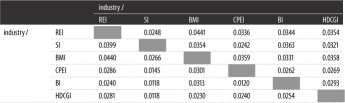

When the threshold of Granger causality is greater than 0.035, there are eight directed edges between industries. It shows the strong Granger causalities between these industries: REI↔BMI, SI→REI, SI→BI, BMI→CPEI, BMI→HDCGI and REI→HDCGI. Among them, there is bidirectional Granger causality between the REI and BMI, and the others are all unidirectional Granger causalities. The results show the diffusion path of the fluctuation between the industries. For example, the SI does not easily Granger cause the HDCGI directly. However, SI may influence HDCGI by other industries. The diffusion paths of the influence may be SI→REI→HDCGI or SI→REI→BMI→HDCGI.

## 4. Discussion and conclusion

In this paper, we built two kinds of network models, a cointegration network model and a Granger causality network model, for understanding the interaction behaviours in the financial stock system. It is helpful to study the long-term equilibrium relationships in the system by the cointegration network model analysis, and it is useful to study the diffusion paths of the fluctuation or the potential risks in the system by the Granger causality network model analysis. The advantage of the network analysis paradigm is that easy availability of the time series data makes the model building time efficient. Moreover, we can understand the risk diffusion of the industrial level from the microstructure perspective. There is some insight for policy decisions and risk diffusion prevention based on the analysis results.

(1) As is known, there are close correlations between the REI and its relative industries. However, the long-term equilibrium relationships are sparse in the financial stock system. The cointegration network shows the equilibrium situation in the system, but only a few stocks play important roles for the equilibrium. We should pay attention to these stocks to a larger degree, because the violent fluctuation from these stocks may lead to a short-term shock.
(2) The cointegration network has good connectivity. This result provides important insights for policy decisions. The REI has a long industry chain and wide influence scope; however, the influence from the fluctuation of any factor can spread conveniently in the system. Based on the difference in the extent of cointegration between industries, decision makers can judge the influence scope of other industries when one industry fluctuates dramatically. For example, when the REI fluctuates dramatically, the BMI and the BI are greatly influenced, but the CPEI is influenced to a relatively weak degree.
(3) The in-degree and out-degree both follow the long-tail distributions in the Granger causality network. It means that there exist a few key stocks that play mainly a driving role in the diffusion process in the financial stock system. Decision makers should pay more attention to these key stocks for risk monitoring. Moreover, there are different types of stocks in the system; some stocks can Granger cause many other stocks, but they are not easily influenced by others, and some stocks are in a contrary situation. These results can help decision makers to identify the role of each stock in risk monitoring.
(4) Based on the difference in the extent of Granger causality between industries, it is helpful to detect the diffusion path of the fluctuation between the industries. In particular, in China, the funds of the REI are mainly dependent on bank loans. The consequences would be ghastly if a financial crisis occurred in China. There would be an unimaginable systematic collapse problem. Thus, to prevent risk diffusion, the results provide very important information for the visualization of the diffusion path. Meanwhile, similar to the control of the spread of a virus, we can monitor and intervene in the transmission medium to curb risk diffusion in the system.

In this paper, we only take the China financial stock system as an empirical sample and mainly focus on the current situation. In the future, we will continue studying the financial stock system of other countries and compare them with each other. We will also study the evolution dynamics of the financial stock system over time.

## Supplementary Material

Table S1

## Supplementary Material

Data

## Data accessibility

The datasets supporting this article have been uploaded as part of the electronic supplementary material.
