# Supplementary material for: Modelling cointegration and Granger causality network to detect long-term equilibrium and diffusion paths in the financial system
Source: R Soc Open Sci. 2018 Mar 28;5(3):172092. doi: 10.1098/rsos.172092 (PMC5882728; doi:10.1098/rsos.172092)
Supplement: Table S1 [file rsos172092supp1.docx]

Table S1 The statistic of each stock

| **No.** | **Stock Code** | **Industry** | **Cointegration** | **Granger causality** | | | |
| --- | --- | --- | --- | --- | --- | --- | --- |
|  |  |  | **Degree** | **In-degree** | **Out-degree** | **Betweenness Centrality** | **Quadrant** |
| 1 | 000558_SZ | REI | 155 | 23 | 90 | 0.003 | I |
| 2 | 000616_SZ | REI | 155 | 11 | 64 | 0.001 | II |
| 3 | 000671_SZ | REI | 155 | 23 | 85 | 0.003 | I |
| 4 | 000926_SZ | REI | 155 | 22 | 63 | 0.001 | I |
| 5 | 000931_SZ | REI | 155 | 22 | 80 | 0.002 | I |
| 6 | 000979_SZ | REI | 155 | 63 | 79 | 0.007 | I |
| 7 | 600223_SH | REI | 155 | 16 | 74 | 0.001 | II |
| 8 | 600466_SH | REI | 155 | 107 | 74 | 0.012 | I |
| 9 | 600565_SH | REI | 155 | 21 | 72 | 0.001 | I |
| 10 | 600665_SH | REI | 155 | 13 | 76 | 0.001 | II |
| 11 | 600716_SH | REI | 155 | 2 | 81 | 0.000 | II |
| 12 | 600724_SH | REI | 155 | 42 | 46 | 0.002 | I |
| 13 | 600895_SH | REI | 155 | 14 | 88 | 0.002 | II |
| 14 | 601588_SH | REI | 155 | 4 | 79 | 0.000 | II |
| 15 | 002233_SZ | BMI | 155 | 13 | 69 | 0.001 | II |
| 16 | 300093_SZ | BMI | 155 | 8 | 104 | 0.002 | II |
| 17 | 002392_SZ | CPEI | 155 | 9 | 81 | 0.001 | II |
| 18 | 600133_SH | CPEI | 155 | 61 | 60 | 0.006 | I |
| 19 | 600284_SH | CPEI | 155 | 26 | 97 | 0.007 | I |
| 20 | 600463_SH | CPEI | 155 | 11 | 89 | 0.001 | II |
| 21 | 601226_SH | CPEI | 155 | 65 | 91 | 0.010 | I |
| 22 | 600015_SH | BI | 155 | 42 | 24 | 0.001 | I |
| 23 | 601988_SH | BI | 155 | 8 | 47 | 0.000 | II |
| 24 | 000882_SZ | REI | 154 | 1 | 95 | 0.000 | II |
| 25 | 600684_SH | REI | 154 | 19 | 55 | 0.001 | II |
| 26 | 600586_SH | BMI | 152 | 37 | 61 | 0.004 | I |
| 27 | 600165_SH | SI | 151 | 41 | 78 | 0.007 | I |
| 28 | 600978_SH | HDCGI | 150 | 38 | 83 | 0.006 | I |
| 29 | 000663_SZ | HDCGI | 149 | 30 | 34 | 0.002 | I |
| 30 | 300135_SZ | BMI | 146 | 52 | 111 | 0.013 | I |
| 31 | 000517_SZ | REI | 145 | 15 | 46 | 0.001 | II |
| 32 | 603969_SH | SI | 144 | 27 | 50 | 0.001 | I |
| 33 | 002375_SZ | CPEI | 143 | 25 | 32 | 0.001 | I |
| 34 | 600696_SH | REI | 141 | 17 | 76 | 0.002 | II |
| 35 | 600067_SH | REI | 140 | 65 | 41 | 0.003 | I |
| 36 | 600683_SH | REI | 140 | 11 | 10 | 0.000 | III |
| 37 | 601969_SH | SI | 140 | 6 | 37 | 0.000 | II |
| 38 | 600307_SH | SI | 139 | 13 | 90 | 0.001 | II |
| 39 | 600399_SH | SI | 139 | 8 | 56 | 0.000 | II |
| 40 | 600010_SH | SI | 128 | 17 | 99 | 0.005 | II |
| 41 | 002116_SZ | CPEI | 127 | 35 | 33 | 0.001 | I |
| 42 | 000667_SZ | REI | 126 | 10 | 59 | 0.001 | II |
| 43 | 600720_SH | BMI | 125 | 46 | 66 | 0.006 | I |
| 44 | 601618_SH | CPEI | 123 | 10 | 53 | 0.001 | II |
| 45 | 000877_SZ | BMI | 122 | 46 | 79 | 0.007 | I |
| 46 | 000029_SZ | REI | 121 | 78 | 35 | 0.007 | I |
| 47 | 000861_SZ | REI | 120 | 15 | 45 | 0.001 | II |
| 48 | 002398_SZ | BMI | 120 | 61 | 26 | 0.001 | I |
| 49 | 600282_SH | SI | 118 | 12 | 49 | 0.001 | II |
| 50 | 600510_SH | REI | 113 | 58 | 25 | 0.001 | I |
| 51 | 600743_SH | REI | 112 | 56 | 53 | 0.004 | I |
| 52 | 000778_SZ | SI | 112 | 18 | 47 | 0.001 | II |
| 53 | 600503_SH | REI | 109 | 62 | 27 | 0.005 | I |
| 54 | 300055_SZ | CPEI | 109 | 47 | 13 | 0.001 | IV |
| 55 | 600708_SH | REI | 108 | 40 | 15 | 0.001 | IV |
| 56 | 600449_SH | BMI | 108 | 19 | 15 | 0.001 | III |
| 57 | 002307_SZ | CPEI | 108 | 36 | 30 | 0.001 | I |
| 58 | 601117_SH | CPEI | 108 | 4 | 31 | 0.001 | II |
| 59 | 000040_SZ | REI | 107 | 43 | 25 | 0.002 | I |
| 60 | 002586_SZ | CPEI | 107 | 0 | 21 | 0.000 | II |
| 61 | 601328_SH | BI | 106 | 45 | 17 | 0.002 | IV |
| 62 | 002084_SZ | HDCGI | 104 | 28 | 13 | 0.001 | IV |
| 63 | 900911_SH | REI | 102 | 33 | 4 | 0.000 | IV |
| 64 | 600641_SH | REI | 102 | 52 | 15 | 0.001 | IV |
| 65 | 000885_SZ | BMI | 102 | 33 | 25 | 0.001 | I |
| 66 | 000540_SZ | REI | 99 | 17 | 23 | 0.000 | II |
| 67 | 002616_SZ | HDCGI | 99 | 54 | 31 | 0.004 | I |
| 68 | 600340_SH | REI | 98 | 45 | 20 | 0.001 | I |
| 69 | 000628_SZ | CPEI | 98 | 33 | 20 | 0.001 | I |
| 70 | 000031_SZ | REI | 97 | 65 | 32 | 0.002 | I |
| 71 | 600246_SH | REI | 97 | 20 | 23 | 0.001 | I |
| 72 | 000498_SZ | CPEI | 97 | 28 | 20 | 0.001 | I |
| 73 | 002163_SZ | CPEI | 97 | 22 | 12 | 0.000 | IV |
| 74 | 000046_SZ | REI | 96 | 53 | 15 | 0.002 | IV |
| 75 | 000012_SZ | BMI | 96 | 63 | 16 | 0.001 | IV |
| 76 | 002671_SZ | CPEI | 93 | 38 | 5 | 0.000 | IV |
| 77 | 002016_SZ | REI | 91 | 13 | 30 | 0.001 | II |
| 78 | 600225_SH | REI | 91 | 21 | 53 | 0.003 | I |
| 79 | 603030_SH | CPEI | 91 | 62 | 17 | 0.001 | IV |
| 80 | 601998_SH | BI | 91 | 17 | 14 | 0.000 | III |
| 81 | 002468_SZ | CPEI | 86 | 2 | 25 | 0.000 | II |
| 82 | 600326_SH | CPEI | 85 | 35 | 14 | 0.000 | IV |
| 83 | 600039_SH | CPEI | 85 | 29 | 21 | 0.000 | I |
| 84 | 002318_SZ | SI | 84 | 42 | 24 | 0.001 | I |
| 85 | 600881_SH | BMI | 83 | 7 | 25 | 0.000 | II |
| 86 | 600846_SH | CPEI | 83 | 57 | 8 | 0.000 | IV |
| 87 | 600082_SH | REI | 81 | 9 | 5 | 0.000 | III |
| 88 | 000672_SZ | BMI | 81 | 5 | 2 | 0.000 | III |
| 89 | 000655_SZ | SI | 79 | 24 | 7 | 0.000 | IV |
| 90 | 601636_SH | BMI | 79 | 13 | 16 | 0.000 | III |
| 91 | 601818_SH | BI | 79 | 39 | 18 | 0.001 | IV |
| 92 | 601939_SH | BI | 79 | 53 | 16 | 0.001 | IV |
| 93 | 600266_SH | REI | 78 | 49 | 21 | 0.001 | I |
| 94 | 000514_SZ | REI | 77 | 23 | 11 | 0.000 | IV |
| 95 | 600208_SH | REI | 77 | 58 | 24 | 0.001 | I |
| 96 | 600532_SH | SI | 77 | 54 | 16 | 0.001 | IV |
| 97 | 002135_SZ | CPEI | 76 | 11 | 39 | 0.002 | II |
| 98 | 600048_SH | REI | 75 | 32 | 4 | 0.000 | IV |
| 99 | 002659_SZ | CPEI | 75 | 54 | 6 | 0.001 | IV |
| 100 | 000809_SZ | CPEI | 75 | 23 | 19 | 0.000 | IV |
| 101 | 002694_SZ | CPEI | 75 | 64 | 38 | 0.002 | I |
| 102 | 002718_SZ | CPEI | 74 | 70 | 33 | 0.002 | I |
| 103 | 600376_SH | REI | 73 | 62 | 31 | 0.001 | I |
| 104 | 300234_SZ | CPEI | 73 | 68 | 25 | 0.001 | I |
| 105 | 600730_SH | REI | 71 | 6 | 11 | 0.000 | III |
| 106 | 002205_SZ | CPEI | 70 | 41 | 19 | 0.000 | IV |
| 107 | 002541_SZ | CPEI | 70 | 40 | 29 | 0.001 | I |
| 108 | 601669_SH | CPEI | 69 | 5 | 21 | 0.000 | II |
| 109 | 000502_SZ | REI | 68 | 8 | 7 | 0.000 | III |
| 110 | 600064_SH | REI | 68 | 56 | 33 | 0.002 | I |
| 111 | 603600_SH | HDCGI | 67 | 36 | 10 | 0.000 | IV |
| 112 | 200029_SZ | REI | 66 | 51 | 25 | 0.004 | I |
| 113 | 000546_SZ | BMI | 66 | 18 | 21 | 0.001 | II |
| 114 | 002457_SZ | CPEI | 65 | 49 | 20 | 0.000 | I |
| 115 | 603818_SH | HDCGI | 65 | 54 | 33 | 0.002 | I |
| 116 | 600240_SH | REI | 64 | 23 | 5 | 0.000 | IV |
| 117 | 600159_SH | REI | 64 | 26 | 10 | 0.000 | IV |
| 118 | 002088_SZ | BMI | 63 | 61 | 33 | 0.002 | I |
| 119 | 600393_SH | REI | 62 | 51 | 26 | 0.001 | I |
| 120 | 000708_SZ | SI | 62 | 25 | 14 | 0.000 | IV |
| 121 | 002596_SZ | BMI | 62 | 51 | 10 | 0.001 | IV |
| 122 | 002443_SZ | SI | 61 | 28 | 19 | 0.001 | IV |
| 123 | 002077_SZ | REI | 60 | 48 | 13 | 0.000 | IV |
| 124 | 000918_SZ | REI | 60 | 55 | 27 | 0.001 | I |
| 125 | 000573_SZ | REI | 59 | 11 | 11 | 0.000 | III |
| 126 | 000786_SZ | BMI | 59 | 40 | 16 | 0.001 | IV |
| 127 | 600239_SH | REI | 58 | 42 | 13 | 0.001 | IV |
| 128 | 000014_SZ | REI | 57 | 42 | 13 | 0.000 | IV |
| 129 | 603008_SH | HDCGI | 57 | 42 | 14 | 0.000 | IV |
| 130 | 000732_SZ | REI | 56 | 20 | 11 | 0.000 | IV |
| 131 | 000619_SZ | CPEI | 56 | 40 | 16 | 0.000 | IV |
| 132 | 000509_SZ | CPEI | 56 | 36 | 16 | 0.000 | IV |
| 133 | 000797_SZ | REI | 55 | 44 | 19 | 0.001 | IV |
| 134 | 600992_SH | SI | 55 | 33 | 14 | 0.000 | IV |
| 135 | 000620_SZ | REI | 54 | 50 | 13 | 0.000 | IV |
| 136 | 600293_SH | BMI | 54 | 37 | 16 | 0.000 | IV |
| 137 | 601398_SH | BI | 54 | 16 | 14 | 0.000 | III |
| 138 | 600769_SH | CPEI | 53 | 38 | 14 | 0.000 | IV |
| 139 | 600621_SH | REI | 52 | 43 | 13 | 0.000 | IV |
| 140 | 002305_SZ | REI | 51 | 26 | 12 | 0.000 | IV |
| 141 | 600658_SH | REI | 51 | 36 | 13 | 0.000 | IV |
| 142 | 600668_SH | BMI | 51 | 50 | 13 | 0.000 | IV |
| 143 | 600802_SH | BMI | 51 | 39 | 11 | 0.000 | IV |
| 144 | 002545_SZ | CPEI | 51 | 28 | 10 | 0.000 | IV |
| 145 | 002524_SZ | CPEI | 50 | 42 | 18 | 0.000 | IV |
| 146 | 000965_SZ | REI | 48 | 40 | 25 | 0.000 | I |
| 147 | 600733_SH | REI | 47 | 33 | 21 | 0.000 | I |
| 148 | 601166_SH | BI | 47 | 16 | 7 | 0.000 | III |
| 149 | 600491_SH | CPEI | 46 | 38 | 24 | 0.000 | I |
| 150 | 000402_SZ | REI | 45 | 34 | 10 | 0.000 | IV |
| 151 | 000609_SZ | REI | 45 | 41 | 16 | 0.000 | IV |
| 152 | 002060_SZ | CPEI | 44 | 28 | 13 | 0.000 | IV |
| 153 | 000042_SZ | REI | 42 | 30 | 13 | 0.000 | IV |
| 154 | 600678_SH | BMI | 41 | 27 | 27 | 0.033 | I |
| 155 | 600095_SH | REI | 39 | 29 | 9 | 0.000 | IV |
| 156 | 600820_SH | CPEI | 38 | 24 | 8 | 0.000 | IV |
| 157 | 002142_SZ | BI | 33 | 30 | 10 | 0.000 | IV |
| 158 | 002162_SZ | CPEI | 32 | 20 | 18 | 0.026 | IV |
| 159 | 600569_SH | SI | 31 | 13 | 22 | 0.011 | II |
| 160 | 002244_SZ | REI | 26 | 11 | 8 | 0.014 | III |
| 161 | 600170_SH | CPEI | 26 | 9 | 13 | 0.009 | III |
| 162 | 000006_SZ | REI | 24 | 16 | 15 | 0.011 | III |
| 163 | 600496_SH | CPEI | 23 | 6 | 12 | 0.009 | III |
| 164 | 002140_SZ | CPEI | 23 | 3 | 23 | 0.010 | II |
| 165 | 000886_SZ | REI | 22 | 16 | 14 | 0.026 | III |
| 166 | 002051_SZ | CPEI | 22 | 8 | 13 | 0.008 | III |
| 167 | 600325_SH | REI | 21 | 20 | 12 | 0.015 | IV |
| 168 | 600022_SH | SI | 21 | 5 | 14 | 0.006 | III |
| 169 | 002133_SZ | REI | 20 | 4 | 9 | 0.001 | III |
| 170 | 600053_SH | REI | 20 | 9 | 12 | 0.005 | III |
| 171 | 002146_SZ | REI | 19 | 8 | 9 | 0.012 | III |
| 172 | 200761_SZ | SI | 19 | 11 | 10 | 0.005 | III |
| 173 | 600777_SH | REI | 18 | 15 | 5 | 0.010 | III |
| 174 | 600639_SH | REI | 18 | 17 | 7 | 0.008 | III |
| 175 | 601390_SH | CPEI | 18 | 8 | 11 | 0.011 | III |
| 176 | 600736_SH | REI | 17 | 12 | 13 | 0.024 | III |
| 177 | 600094_SH | REI | 17 | 13 | 9 | 0.013 | III |
| 178 | 000935_SZ | BMI | 17 | 9 | 10 | 0.008 | III |
| 179 | 600000_SH | BI | 17 | 11 | 6 | 0.006 | III |
| 180 | 000897_SZ | REI | 16 | 2 | 11 | 0.001 | III |
| 181 | 200011_SZ | REI | 16 | 4 | 14 | 0.002 | III |
| 182 | 000631_SZ | REI | 16 | 13 | 7 | 0.005 | III |
| 183 | 600019_SH | SI | 16 | 8 | 9 | 0.004 | III |
| 184 | 002743_SZ | CPEI | 16 | 9 | 8 | 0.001 | III |
| 185 | 600533_SH | REI | 15 | 8 | 0 | 0.000 | III |
| 186 | 002285_SZ | REI | 15 | 8 | 11 | 0.009 | III |
| 187 | 000838_SZ | REI | 15 | 3 | 14 | 0.006 | III |
| 188 | 600808_SH | SI | 15 | 5 | 8 | 0.001 | III |
| 189 | 002756_SZ | SI | 15 | 10 | 5 | 0.003 | III |
| 190 | 603017_SH | CPEI | 15 | 7 | 9 | 0.004 | III |
| 191 | 900902_SH | REI | 14 | 7 | 3 | 0.006 | III |
| 192 | 600077_SH | REI | 14 | 13 | 9 | 0.002 | III |
| 193 | 000709_SZ | SI | 14 | 12 | 4 | 0.003 | III |
| 194 | 000825_SZ | SI | 14 | 9 | 8 | 0.004 | III |
| 195 | 200018_SZ | CPEI | 14 | 6 | 10 | 0.004 | III |
| 196 | 000001_SZ | BI | 14 | 7 | 4 | 0.008 | III |
| 197 | 601009_SH | BI | 14 | 8 | 7 | 0.003 | III |
| 198 | 300089_SZ | HDCGI | 14 | 8 | 7 | 0.001 | III |
| 199 | 000011_SZ | REI | 13 | 8 | 7 | 0.003 | III |
| 200 | 600162_SH | REI | 13 | 9 | 6 | 0.006 | III |
| 201 | 002302_SZ | BMI | 13 | 5 | 9 | 0.002 | III |
| 202 | 000018_SZ | CPEI | 13 | 6 | 7 | 0.002 | III |
| 203 | 002403_SZ | HDCGI | 13 | 13 | 3 | 0.009 | III |
| 204 | 600234_SH | REI | 12 | 6 | 12 | 0.009 | III |
| 205 | 600052_SH | REI | 12 | 9 | 5 | 0.006 | III |
| 206 | 600005_SH | SI | 12 | 7 | 11 | 0.004 | III |
| 207 | 000898_SZ | SI | 12 | 5 | 5 | 0.001 | III |
| 208 | 002062_SZ | CPEI | 12 | 3 | 8 | 0.004 | III |
| 209 | 600970_SH | CPEI | 12 | 8 | 5 | 0.000 | III |
| 210 | 000090_SZ | CPEI | 12 | 8 | 5 | 0.004 | III |
| 211 | 600528_SH | CPEI | 12 | 3 | 3 | 0.000 | III |
| 212 | 603979_SH | CPEI | 12 | 11 | 5 | 0.007 | III |
| 213 | 600185_SH | REI | 11 | 4 | 7 | 0.000 | III |
| 214 | 000056_SZ | REI | 11 | 6 | 5 | 0.003 | III |
| 215 | 300409_SZ | BMI | 11 | 6 | 5 | 0.002 | III |
| 216 | 002247_SZ | CPEI | 11 | 2 | 10 | 0.002 | III |
| 217 | 002081_SZ | CPEI | 11 | 3 | 8 | 0.003 | III |
| 218 | 002323_SZ | CPEI | 11 | 4 | 7 | 0.001 | III |
| 219 | 000615_SZ | REI | 10 | 5 | 6 | 0.001 | III |
| 220 | 600823_SH | REI | 10 | 5 | 5 | 0.000 | III |
| 221 | 600400_SH | REI | 10 | 4 | 10 | 0.005 | III |
| 222 | 600117_SH | SI | 10 | 3 | 2 | 0.000 | III |
| 223 | 002352_SZ | SI | 10 | 9 | 7 | 0.003 | III |
| 224 | 601992_SH | BMI | 10 | 5 | 7 | 0.001 | III |
| 225 | 000961_SZ | CPEI | 10 | 5 | 5 | 0.000 | III |
| 226 | 002482_SZ | CPEI | 10 | 6 | 4 | 0.001 | III |
| 227 | 002469_SZ | CPEI | 10 | 5 | 5 | 0.002 | III |
| 228 | 600193_SH | CPEI | 10 | 6 | 6 | 0.004 | III |
| 229 | 600383_SH | REI | 9 | 6 | 3 | 0.002 | III |
| 230 | 600791_SH | REI | 9 | 2 | 3 | 0.000 | III |
| 231 | 600007_SH | REI | 9 | 9 | 3 | 0.006 | III |
| 232 | 200056_SZ | REI | 9 | 4 | 4 | 0.001 | III |
| 233 | 600231_SH | SI | 9 | 4 | 6 | 0.000 | III |
| 234 | 000789_SZ | BMI | 9 | 5 | 5 | 0.000 | III |
| 235 | 600425_SH | BMI | 9 | 4 | 1 | 0.001 | III |
| 236 | 002504_SZ | CPEI | 9 | 5 | 5 | 0.001 | III |
| 237 | 603616_SH | CPEI | 9 | 7 | 3 | 0.008 | III |
| 238 | 600883_SH | BMI | 8 | 5 | 4 | 0.002 | III |
| 239 | 601186_SH | CPEI | 8 | 5 | 5 | 0.009 | III |
| 240 | 300117_SZ | CPEI | 8 | 2 | 7 | 0.000 | III |
| 241 | 601288_SH | BI | 8 | 3 | 5 | 0.001 | III |
| 242 | 600016_SH | BI | 8 | 4 | 5 | 0.002 | III |
| 243 | 600036_SH | BI | 8 | 6 | 1 | 0.000 | III |
| 244 | 002147_SZ | REI | 7 | 3 | 3 | 0.002 | III |
| 245 | 000656_SZ | REI | 7 | 4 | 5 | 0.003 | III |
| 246 | 000863_SZ | REI | 7 | 6 | 3 | 0.002 | III |
| 247 | 000567_SZ | REI | 7 | 2 | 1 | 0.000 | III |
| 248 | 600657_SH | REI | 7 | 5 | 2 | 0.002 | III |
| 249 | 000959_SZ | SI | 7 | 5 | 6 | 0.001 | III |
| 250 | 600801_SH | BMI | 7 | 7 | 3 | 0.002 | III |
| 251 | 000928_SZ | CPEI | 7 | 3 | 3 | 0.000 | III |
| 252 | 002333_SZ | CPEI | 7 | 4 | 5 | 0.001 | III |
| 253 | 002713_SZ | CPEI | 7 | 5 | 4 | 0.004 | III |
| 254 | 002032_SZ | HDCGI | 7 | 3 | 7 | 0.005 | III |
| 255 | 600638_SH | REI | 6 | 5 | 3 | 0.002 | III |
| 256 | 600807_SH | REI | 6 | 1 | 4 | 0.005 | III |
| 257 | 600126_SH | SI | 6 | 3 | 6 | 0.001 | III |
| 258 | 002132_SZ | SI | 6 | 3 | 4 | 0.001 | III |
| 259 | 000761_SZ | SI | 6 | 1 | 5 | 0.000 | III |
| 260 | 600248_SH | CPEI | 6 | 4 | 3 | 0.002 | III |
| 261 | 002047_SZ | CPEI | 6 | 0 | 4 | 0.000 | III |
| 262 | 600692_SH | REI | 5 | 5 | 0 | 0.000 | III |
| 263 | 002208_SZ | REI | 5 | 4 | 3 | 0.004 | III |
| 264 | 000909_SZ | REI | 5 | 5 | 1 | 0.000 | III |
| 265 | 000890_SZ | SI | 5 | 4 | 4 | 0.002 | III |
| 266 | 600819_SH | BMI | 5 | 3 | 2 | 0.002 | III |
| 267 | 002628_SZ | CPEI | 5 | 4 | 3 | 0.003 | III |
| 268 | 601800_SH | CPEI | 5 | 4 | 1 | 0.000 | III |
| 269 | 300197_SZ | CPEI | 5 | 5 | 1 | 0.001 | III |
| 270 | 601886_SH | CPEI | 5 | 2 | 1 | 0.000 | III |
| 271 | 300198_SZ | CPEI | 5 | 3 | 3 | 0.002 | III |
| 272 | 002325_SZ | CPEI | 5 | 1 | 3 | 0.000 | III |
| 273 | 601169_SH | BI | 5 | 2 | 4 | 0.002 | III |
| 274 | 000036_SZ | REI | 4 | 3 | 1 | 0.000 | III |
| 275 | 002271_SZ | BMI | 4 | 4 | 2 | 0.000 | III |
| 276 | 900957_SH | BMI | 4 | 4 | 1 | 0.001 | III |
| 277 | 002372_SZ | CPEI | 4 | 2 | 1 | 0.002 | III |
| 278 | 600083_SH | CPEI | 4 | 1 | 3 | 0.000 | III |
| 279 | 000055_SZ | CPEI | 4 | 3 | 3 | 0.003 | III |
| 280 | 900939_SH | HDCGI | 4 | 4 | 1 | 0.001 | III |
| 281 | 002572_SZ | HDCGI | 4 | 1 | 4 | 0.000 | III |
| 282 | 000668_SZ | REI | 3 | 2 | 1 | 0.000 | III |
| 283 | 000691_SZ | REI | 3 | 1 | 2 | 0.002 | III |
| 284 | 000981_SZ | REI | 3 | 0 | 3 | 0.000 | III |
| 285 | 600606_SH | REI | 3 | 2 | 3 | 0.002 | III |
| 286 | 200012_SZ | BMI | 3 | 0 | 0 | 0.000 | III |
| 287 | 600585_SH | BMI | 3 | 3 | 2 | 0.000 | III |
| 288 | 600477_SH | CPEI | 3 | 1 | 3 | 0.000 | III |
| 289 | 600853_SH | CPEI | 3 | 3 | 1 | 0.000 | III |
| 290 | 600068_SH | CPEI | 3 | 3 | 2 | 0.001 | III |
| 291 | 200055_SZ | CPEI | 3 | 3 | 1 | 0.000 | III |
| 292 | 002615_SZ | HDCGI | 3 | 2 | 1 | 0.000 | III |
| 293 | 002120_SZ | HDCGI | 3 | 3 | 0 | 0.000 | III |
| 294 | 002035_SZ | HDCGI | 3 | 2 | 1 | 0.000 | III |
| 295 | 600604_SH | REI | 2 | 2 | 1 | 0.000 | III |
| 296 | 600322_SH | REI | 2 | 1 | 2 | 0.000 | III |
| 297 | 600622_SH | REI | 2 | 2 | 0 | 0.000 | III |
| 298 | 601003_SH | SI | 2 | 0 | 2 | 0.000 | III |
| 299 | 002755_SZ | CPEI | 2 | 1 | 1 | 0.000 | III |
| 300 | 600748_SH | REI | 1 | 0 | 1 | 0.000 | III |
| 301 | 002113_SZ | REI | 1 | 1 | 1 | 0.000 | III |
| 302 | 000711_SZ | REI | 1 | 0 | 1 | 0.000 | III |
| 303 | 600608_SH | SI | 1 | 1 | 1 | 0.000 | III |
| 304 | 600507_SH | SI | 1 | 1 | 0 | 0.000 | III |
| 305 | 600782_SH | SI | 1 | 1 | 0 | 0.000 | III |
| 306 | 601028_SH | SI | 1 | 1 | 1 | 0.000 | III |
| 307 | 600512_SH | CPEI | 1 | 1 | 0 | 0.000 | III |
| 308 | 000065_SZ | CPEI | 1 | 1 | 0 | 0.000 | III |
| 309 | 603898_SH | HDCGI | 1 | 1 | 1 | 0.000 | III |
